# Iron insertion into coproporphyrin III‐ferrochelatase complex: Evidence for an intermediate distorted catalytic species

**DOI:** 10.1002/pro.4788

**Published:** 2023-10-16

**Authors:** Thomas Gabler, Andrea Dali, Federico Sebastiani, Paul Georg Furtmüller, Maurizio Becucci, Stefan Hofbauer, Giulietta Smulevich

**Affiliations:** ^1^ Department of Chemistry Institute of Biochemistry, University of Natural Resources and Life Sciences Vienna Austria; ^2^ Dipartimento di Chimica “Ugo Schiff”—DICUS Università di Firenze Sesto Fiorentino Italy; ^3^ INSTM Research Unit of Firenze Sesto Fiorentino Italy

**Keywords:** crystal soaking, ferrochelatase, metal titration, metalation, resonance Raman spectroscopy, x‐ray crystallography

## Abstract

Understanding the reaction mechanism of enzymes at the molecular level is generally a difficult task, since many parameters affect the turnover. Often, due to high reactivity and formation of transient species or intermediates, detailed information on enzymatic catalysis is obtained by means of model substrates. Whenever possible, it is essential to confirm a reaction mechanism based on substrate analogues or model systems by using the physiological substrates. Here we disclose the ferrous iron incorporation mechanism, in solution, and in crystallo, by the coproporphyrin III‐coproporphyrin ferrochelatase complex from the firmicute, pathogen, and antibiotic resistant, *Listeria monocytogenes*. Coproporphyrin ferrochelatase plays an important physiological role as the metalation represents the penultimate reaction step in the prokaryotic coproporphyrin‐dependent heme biosynthetic pathway, yielding coproheme (ferric coproporphyrin III). By following the metal titration with resonance Raman spectroscopy and x‐ray crystallography, we prove that upon metalation the saddling distortion becomes predominant both in the crystal and in solution. This is a consequence of the readjustment of hydrogen bond interactions of the propionates with the protein scaffold during the enzymatic catalysis. Once the propionates have established the interactions typical of the coproheme complex, the distortion slowly decreases, to reach the almost planar final product.

## 1 INTRODUCTION

Chelatases are a class of enzymes that catalyze the insertion of metal ions into porphyrins. The insertion of ferrous iron into protoporphyrin IX (PPIX) by protoporphyrin ferrochelatase (PpfC) is the terminal step to obtain heme *b* in the protoporphyrin‐dependent (PPD) biosynthetic pathway. Diderm bacteria and eukaryotes predominantly use this well‐characterized PPD pathway, whereas it has been discovered in 2015 that monoderm bacteria utilize a coproporphyrin‐dependent (CPD) pathway (Dailey et al., 2017; Dailey & Gerdes, 2015; Layer, 2021). Coproporphyrin ferrochelatase (CpfC) in the CPD heme biosynthesis pathway incorporates ferrous iron into coproporphyrin III (cpIII) to give coproheme, which is eventually decarboxylated to yield heme *b* by the coproheme decarboxylase enzyme in the final step of this biosynthetic process (Dailey et al., 2017; Dailey & Gerdes, 2015; Layer, 2021).

A comprehensive understanding of the CPD pathway is highly important since bacterial pathogens require heme to cause disease. Understanding structure–function relationships of key enzymes, such as ferrochelatase, is an essential precondition for the design of new inhibitors for antibiotic‐resistant pathogens, like *Listeria monocytogenes*.

The porphyrin metalation reaction of ferrochelatases has been studied for more than three decades (Hunter & Ferreira, 2022; Senge et al., 2015). The general reaction mechanism includes several steps, involving the binding of the porphyrin and the metal ion to the enzyme, deformation of the porphyrin, deprotonation of the porphyrin nitrogens, and metal–ligand exchange with the release of the heme product. A saddling distortion of the porphyrin macrocycle has long been recognized to be a critical step, since it facilitates metal chelation by exposing the lone pair orbitals of the pyrrole nitrogen atoms to the incoming metal ion and facilitates the formation of the first metal–nitrogen bond (Al‐Karadaghi et al., 1997; Beas et al., 2022; Dailey et al., 2000; Hansson et al., 2007; Hansson et al., 2011; Hunter & Ferreira, 2022; Medlock et al., 2021; Wang & Shen, 2013; Wu et al., 2016). Furthermore, the distortion of the tetrapyrrole was found to lead to a higher affinity for the ferrochelatases (Dailey et al., 1989; Dailey & Fleming, 1983; De Matteis, Gibbs, & Smith, 1980; De Matteis, Gibbs, & Tephly, 1980; Wu et al., 2001) and suggested to modulate their metal specificity (Al‐Karadaghi et al., 2006). On the contrary, a lower saddled distortion was associated to a reduced catalytic efficiency as compared to that of the wild‐type (WT) enzyme (Karlberg et al., 2002).

The idea that macrocycle deformation had a pivotal role in the metal insertion process was originally based on the observation that distorted *N*‐alkylporphyrins underwent metalation 3–5 orders of magnitude faster than non‐methylated planar derivatives (Takeda et al., 1992). This hypothesis was subsequently supported by the synthesis of an antibody metalation catalyst, that is, *N*‐methyl mesoporphyrin IX (N‐MeMP) (Cochran & Schultz, 1990). The crystal structure of *B. subtilis* ferrochelatase (*Bs*CpfC) with bound N‐MeMP has served as the basis of mechanistic models for prokaryotic ferrochelatases (Karlberg et al., 2008; Lecerof et al., 2000). It has been postulated that not only the orientation of the N‐MeMP, as observed in the crystal structure, should correspond to that of the natural substrate/product, but also that the macrocycle distortion represents a catalytic intermediate that occurs during normal turnover. However, the spatial position of PPIX in the active site of human ferrochelatase (Medlock et al., 2007) as well as the substrate—(cpIII‐) and product—(coproheme‐) bound to the bacterial firmicute *L. monocytogenes* ferrochelatase *Lm*CpfC (Dali et al., 2023; Hofbauer et al., 2020) revealed a significantly different binding pose than N‐MeMP in *Bs*CpfC, clearly indicating that for each ferrochelatase the physiological substrate needs to be taken into consideration.

Several resonance Raman (RR) spectroscopy studies on the porphyrin binding to murine and human ferrochelatase suggested that the saddle distortion of the macrocycle may derive from the binding of the tetrapyrrole to the active site of the enzyme (Franco et al., 2000; Franco et al., 2011; Karlberg et al., 2002; Lu et al., 2002). In fact, a strong *γ*_15_ out‐of‐plane mode, characteristic of saddling‐like distortions, was activated upon protein binding (Li et al., 1989). Furthermore, it was shown that a lower saddled distortion induced not only a reduced catalytic efficiency, but also alterations in the vibrational modes associated with the porphyrin vinyl and propionate groups, suggesting that a reorientation and relocation of the macrocycle can occur in proteins with mutated active site residues (Shi et al., 2006). RR studies also demonstrated that the interaction of ferrochelatase with a tetrapyrrole and a catalytic antibody, known to catalyze porphyrin metalation, induced different types of distortion, while the activation of the Raman band *γ*_15_, resulting from the out‐of‐plane vibration, was related directly to the degree of affinity maturation of the antibody (Blackwood Jr. et al., 1998; Jarzecki & Spiro, 2005; Venkateshrao et al., 2004). These results suggested that, to a great extent, it is the environment of the active site that affects the orientation and distortion of the porphyrin.

We have recently shown that the substrate cpIII bound to *Lm*CpfC presents a doming deformation in solution and a saddling distortion in the crystal structure (Dali et al., 2023), hence, distinct from the planar coproheme product complex (Dali et al., 2023). Therefore, we proposed that the porphyrin deformation observed upon binding, results from characteristic interactions between the substrate and the protein itself, and that the environment of the active site controls both the orientation and distortion of the porphyrin. This conclusion is in agreement with x‐ray studies on human‐PpfC, which showed that the bound porphyrin macrocycle was only modestly distorted (Medlock et al., 2007), allowing us to suggest that the further saddling predicted to occur upon metalation is reasonably a successive, but not necessarily correlated, step.

To gain insight into the enzymatic mechanism of CpfCs, we devised a comprehensive biochemical, crystallographic, and spectroscopic analysis of *Lm*CpfC in its substrate‐bound form (cpIII), while forming the product‐bound state with iron coproporphyrin III (coproheme) upon metalation. Here we present the results of the Fe^2+^ in vitro titration of the wild‐type (WT) *Lm*CpfC‐cpIII complex followed by x‐ray diffraction of crystals soaked in mother liquor containing 1 mM Fe^2+^ (with various soaking incubation times) and in solution by UV–vis and RR spectroscopies. All together, these results demonstrate a reorientation and relocation of the propionate groups during metalation and deliver a clear picture of the porphyrin saddling distortions, which represent an intermediate catalytic species that occurs during the enzymatic process. Without relying on model systems, but using for the first time the cpIII physiological substrate, we prove that the environment of the active site together with the propionate hydrogen bonding largely control the orientation and distortion of the porphyrin both before and during catalysis.

## 2 RESULTS

### 2.1 Iron insertion followed by x‐ray crystallography

In this study we report three x‐ray crystal structures of *Lm*CpfC during turnover. *Lm*CpfC crystals in complex with cpIII were soaked with a ferrous iron solution and flash‐vitrified after 2, 3, and 4 min, to obtain snapshots of the enzymatic reaction during iron insertion into the porphyrin macrocycle. The experiment allowed us to verify that *Lm*CpfC is active in its crystalline state, even though reaction kinetics presumably are slowed down compared to in‐solution approaches (Schmidt, 2020; Schmidt & Saldin, 2014), due to limited mass exchange rate. The overall structures of the “24%” (soaking time: 2 min), “34%” (soaking time: 3 min), and “68%” (soaking time: 4 min) iron occupancy samples are identical to those of *Lm*CpfC‐cpIII (PDB ID: 8AT8) (Dali et al., 2023) and *Lm*CpfC‐coproheme (PDB ID: 6SV3) (Hofbauer et al., 2020). In fact, their secondary structural elements are perfectly superimposable: the “24% Fe” structure shows an rmsd‐value of the backbone carbons compared to the *Lm*CpfC‐cpIII structure of 0.311 Å and 0.328 Å when aligned with the *Lm*CpfC‐coproheme structure; the “34% Fe” structure shows rmsd‐values of 0.165 Å (compared to *Lm*CpfC‐cpIII) and 0.186 Å (compared to *Lm*CpfC‐coproheme); the “68% Fe” structure shows rmsd‐values of 0.164 Å (compared to *Lm*CpfC‐cpIII) and 0.183 Å (compared to *Lm*CpfC‐coproheme).

In comparison to the *Lm*CpfC‐cpIII complex, specific and marked differences are observed in the active site upon soaking with a ferrous iron solution. In particular, the most prominent changes are observed in (i) the extent of porphyrin distortion, (ii) the occupancy of the ferrous iron inserted during the reaction, and (iii) the interactions between propionates 6 and 7 (p6 and p7) with two arginine residues of the protein cavity (R45—p6; R29—p7).

**FIGURE 1:**
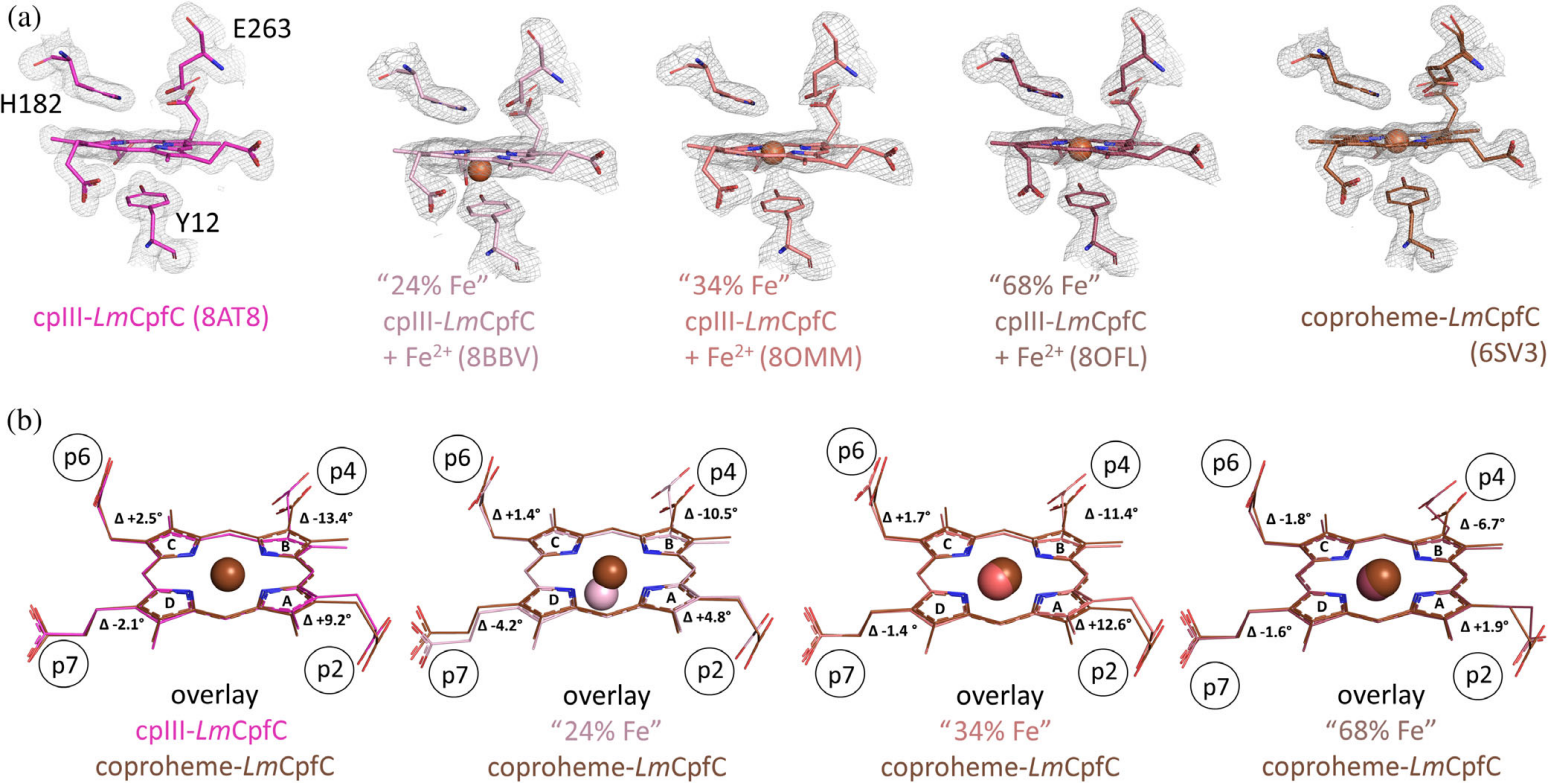
Comparison of the active sites of *Lm*CpfC wild‐type in complex with cpIII (PDB‐ID: 8AT8, pink), with 24% iron occupancy (PDB‐ID: 8BBV, light pink), 34% iron occupancy (pdb‐ID: 8OMM, salmon) and 68% iron occupancy (pdb‐ID: 8OFL, ruby) after ferrous iron soaking. The structure of *Lm*CpfC‐coproheme (PDB‐ID: 6SV3) is depicted in brown. Electron density maps (2fo‐fc) are presented as gray meshes (*σ* = 1.2). (a) Representation of the distal and proximal sides, as well as the porphyrin macrocycle during the iron insertion process. (b) Comparison of pyrrole distortion of all discussed structures.

In the snapshots of the three stages of the catalytic reaction, a clear electron density with different intensity is found in the center of the porphyrin macrocycle, absent in the cpIII structure (PDB ID: 8AT8) and clearly identified in the coproheme structure (PDB ID: 6SV3). In the “24% Fe” structure (resolution 2.2 Å, see data collection and refinement statistics in Table 1) this electron density is found close to pyrrole rings C and D and slightly out of the porphyrin plane toward the proximal tyrosine. Best statistics were obtained when the ferrous iron was placed into the density with an occupancy of 24%. In the “34% Fe” structure (resolution 2.15 Å, Table 1) best statistics were obtained with a 34% iron occupancy fitted into the electron density, which is located slightly toward pyrrole ring C and D and now in plane with the substrate. In the "68% Fe" structure (resolution 2.1 Å, Table 1) the best refinement solution was identified with the ferrous iron being present at 68% occupancy. The electron densities (*σ* = 1.2) span from the distal histidine (H182) via the iron of the porphyrin to the proximal tyrosine (Y12) (Figure 1a). The data show that the extent of the porphyrin distortion varies from the substrate—(cpIII‐) bound structure to the product—(coproheme‐) bound structure. The substrate‐bound structure exhibits a saddled porphyrin, whereas the coproheme structure is almost completely planar. During the reaction different degrees of porphyrin distortion are observed in the snapshots (Figure 1b). To better analyze the differences in the tilting of the pyrrole rings we pair‐fitted the four pyrrole nitrogen atoms of the planar coproheme structure to all the other four structures and measured the individual distortion angles of each pyrrole ring (Figure 1b). Ring A with the substituent p2 exhibits the highest distortion in the “34% Fe” structure (+12.6°), +9.2° in the cpIII structure and lowered values for the “24% Fe” (+4.8°), and the “68% Fe” (+1.9°) structures; in ring B (p4) the angle is altered the most in the analyzed samples (−13.4°, −10.5°, −11.4°, and −6.7°) as a function of the site occupancy by Fe ions, exhibiting a high degree of distortion compared to the coproheme structure. In ring C (p6), the distortion variation is as follows, with observed angles of +2.5°, +1.4°, +1.7, and −1.8°. The measured change of angles throughout the ferrous iron insertion process at the site of p7 at pyrrole ring D is also peculiar: the rings in the substrate‐ and product‐bound states overlap well for all structures besides the “24% Fe” structure (−2.1° in the cpIII‐structure, −1.6° in the "68% Fe" structure, and −1.4° in the "34% Fe" structure). Interestingly, in the “24% Fe” structure ring D is significantly more tilted (−4.2°) (Figure S1).

**TABLE 1:** Data collection and refinement statistics.

	“24% Fe‐occupancy” (PDB‐ID: 8BBV)	“34% Fe‐occupancy” (PDB‐ID:8OMM)	“68% Fe‐occupancy” (PDB‐ID: 8OFL)
Resolution range	34.3–2.19 (2.268–2.19)	36.17–2.15 (2.227–2.15)	45.55–2.1 (2.175–2.1)
Space group	*P* 1 21 1	*P* 1 21 1	*P* 1 21 1
Unit cell	36.615 66.919 61.802 90 103.218 90	37.158 67.73 62.494 90 103.221 90	37.439 67.638 63.178 90 102.742 90
Total reflections	29,944 (2988)	32,898 (3264)	35,936 (3513)
Unique reflections	15,035 (1502)	16,505 (1637)	18,016 (1760)
Multiplicity	2.0 (2.0)	2.0 (2.0)	2.0 (2.0)
Completeness (%)	99.79 (99.87)	99.88 (99.94)	99.66 (99.49)
Mean I/sigma (I)	7.50 (1.30)	9.46 (1.52)	10.54 (1.54)
Wilson *B*‐factor	41.76	37.78	39.36
*R*‐merge	0.0613 (0.6036)	0.05183 (0.4136)	0.04134 (0.4567)
*R*‐meas	0.08669 (0.8537)	0.0733 (0.5849)	0.05846 (0.6459)
*R*‐pim	0.0613 (0.6036)	0.05183 (0.4136)	0.04134 (0.4567)
CC1/2	0.996 (0.583)	0.997 (0.792)	0.998 (0.812)
CC*	0.999 (0.858)	0.999 (0.94)	1 (0.947)
Reflections used in refinement	15,025 (1501)	16,493 (1637)	17,973 (1752)
Reflections used for *R*‐free	740 (80)	798 (80)	878 (72)
*R*‐work	0.1944 (0.2955)	0.1817 (0.2937)	0.1802 (0.2822)
*R*‐free	0.2444 (0.3706)	0.2329 (0.3397)	0.2286 (0.3182)
CC (work)	0.956 (0.805)	0.970 (0.896)	0.963 (0.890)
CC (free)	0.947 (0.781)	0.931 (0.885)	0.945 (0.868)
Number of non‐hydrogen atoms	2586	2622	2611
Macromolecules	2489	2491	2491
Ligands	112	114	76
Solvent	33	65	53
Buffer molecules	78	48	22
Porphyrin	1	1	1
Protein residues	307	307	306
RMS (bonds)	0.003	0.002	0.003
RMS (angles)	0.54	0.54	0.76
Ramachandran favored (%)	96.07	97.38	96.38
Ramachandran allowed (%)	3.93	2.62	3.62
Ramachandran outliers (%)	0.00	0.00	0.00
Rotamer outliers (%)	0.76	0.38	0.76
Clashscore	2.41	5.21	7.02
Average *B*‐factor	50.16	44.03	52.24
Macromolecules	50.25	43.95	52.28
Porphyrin ligand	45.52	45.09	46.95
Ligands	47.87	48.44	55.91
Solvent	47.13	42.38	45.90
Number of TLS groups	7	9	5

*Note*: Statistics for the highest‐resolution shell are shown in parentheses. *Structure coordinates*: Coordinates for the cpIII‐*Lm*CpfC wild‐type with 24%‐, 34%‐, and 68%‐Fe occupancy are deposited in the Protein Data Bank (www.pdb.org) and can be found with the accession code 8BBV, 8OMM, and 8OFL, respectively.

**FIGURE 2:**
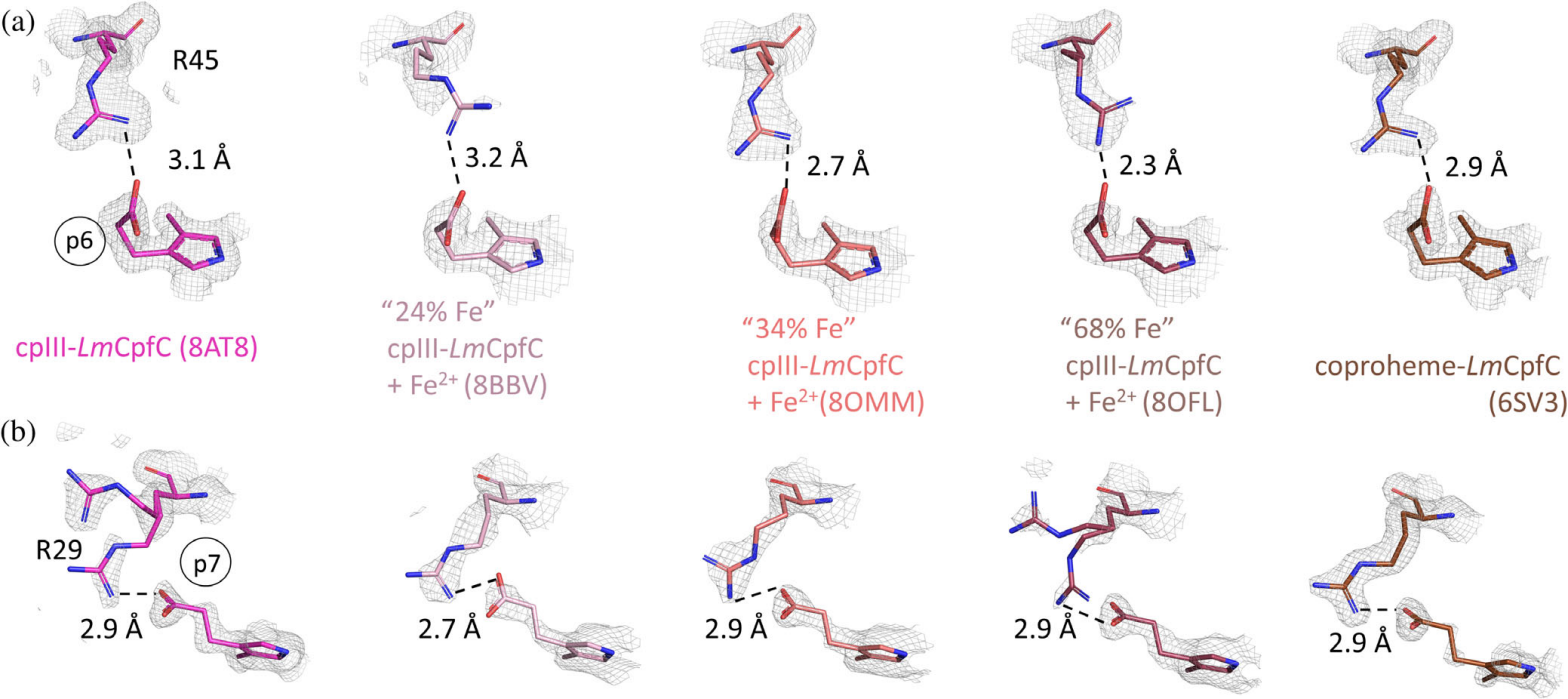
Structural environment of propionates at positions 6 (a) and 7 (b) in *Lm*CpfC wild‐type in complex with cpIII (PDB‐ID: 8AT8, pink), at 24% iron occupancy (PDB‐ID: 8BBV, light pink), at 34% iron occupancy (PDB‐ID: 8OMM, salmon) and at 68% iron occupancy (PDB‐ID: 8OFL, ruby) upon soaking with a ferrous iron solution. The structure of *Lm*CpfC‐coproheme (PDB‐ID: 6SV3) is depicted in brown. Electron density maps (2fo‐fc) are presented as gray meshes (*σ* = 1.2). Distances between the R45 (top) and R29 (bottom) residues, and p6 and p7, respectively, are shown as black dashed lines.

According to the structural data, the hydrogen‐bond interactions involving p2 and p4 do not significantly change during the iron insertion reaction. On the other hand, there are a lot of structural adjustments close to p6 and p7. The distance between the R45 and p6 is always between 2.3 and 3.2 Å for all structures, but this residue is found in different positions (Figure 2a). Therefore, in contrast to the substrate‐ and product‐bound crystals, the two structures representing the on‐going iron insertion show that R45 adopts different conformations during metalation.

In regard to the R29‐p7 H‐bond interaction, it has been already observed that this H‐bond is not always present, since in the *Lm*CpfC‐cpIII crystal structure the R29 residue shows two possible conformations, in and out (Figure 2b and Dali et al., 2023). In solution, the R29L variant shows wild‐type‐like characteristics upon coproheme binding, while upon cpIII binding it shares similarities with the R45L variant, where only the H‐bond with p6 is removed (Dali et al., 2023; Gabler et al., 2022). In this work, changes in the orientation of the R29 residue are observed along the metalation process. In the cpIII‐bound crystal structure the R29 residue is in the split conformation. In the “24% iron occupancy” and in the coproheme‐bound structures, the guanidinium group points toward p7, while in the “68% iron occupancy” structure, being in a split conformation, partially points outwards since one conformation is 5.4 Å from p7 (Figure 2b). Therefore, the overall picture emphasizes the conformational flexibility of this residue in general and especially during turnover.

### 2.2 Iron insertion followed by UV–vis and RR spectroscopies

The UV–vis spectra of cpIII and coproheme complexed with the protein are quite different. In fact, porphyrins have unique UV–vis absorption spectra which do not depend only on the porphyrin structure itself, but also on the ligation of the central metal ion and the chemical environment (Gouterman, 1979). Analogously, RR spectral features of WT *Lm*CpfC‐cpIII and coproheme complexes revealed marked differences, in agreement with their crystal structures (Dali et al., 2023; Gabler et al., 2022). In particular, we have previously shown that insertion of cpIII into the protein gives rise to RR core size marker bands similar to those of the hexacoordinated high spin (6cHS) metallo‐protein species, while the WT‐coproheme complex is characterized by core size marker bands typical of a Fe–Tyr pentacoordinate high spin (5cHS) species (i.e., with a similar intensity of the *ν*_3_ and *ν*_4_ modes). Another substantial difference between the substrate and the product is the presence in the WT‐cpIII of the *γ*_7_, *γ*_6_, out‐of‐plane modes, at 310 and 348 cm^−1^ (Dali et al., 2023). These latter bands, normally RR‐inactive in a planar system, are activated by a doming‐like distortion of the porphyrin ring (Czernuszewicz et al., 1989). This distortion is a consequence of the multiple strong H‐bonds established between the polar side chains of the pocket and the propionate groups of cpIII (Dali et al., 2023). Unlike the WT‐cpIII complex, the insertion of coproheme into the protein does not cause any distortion, and in agreement with the crystal structure (Hofbauer et al., 2020), the porphyrin ring is almost flat. Finally, in the low frequency region, four propionate *δ*(C_β_C_c_C_d_) bending modes were identified in both the WT‐cpIII and coproheme complexes at slightly different wavenumbers (see below) (Dali et al., 2023; Gabler et al., 2022; Sebastiani et al., 2022). As the propionate H‐bonds contribute also to the stabilization and correct orientation of the substrate/product in the active site (Dali et al., 2023; Hofbauer et al., 2020), the different wavenumbers are a consequence of the different strength of their interactions with polar amino acids in the active site. In fact, the stronger the H‐bond interactions, the higher are the propionate bending mode wavenumbers (Gottfried et al., 1996; Peterson et al., 1998).

**FIGURE 3:**
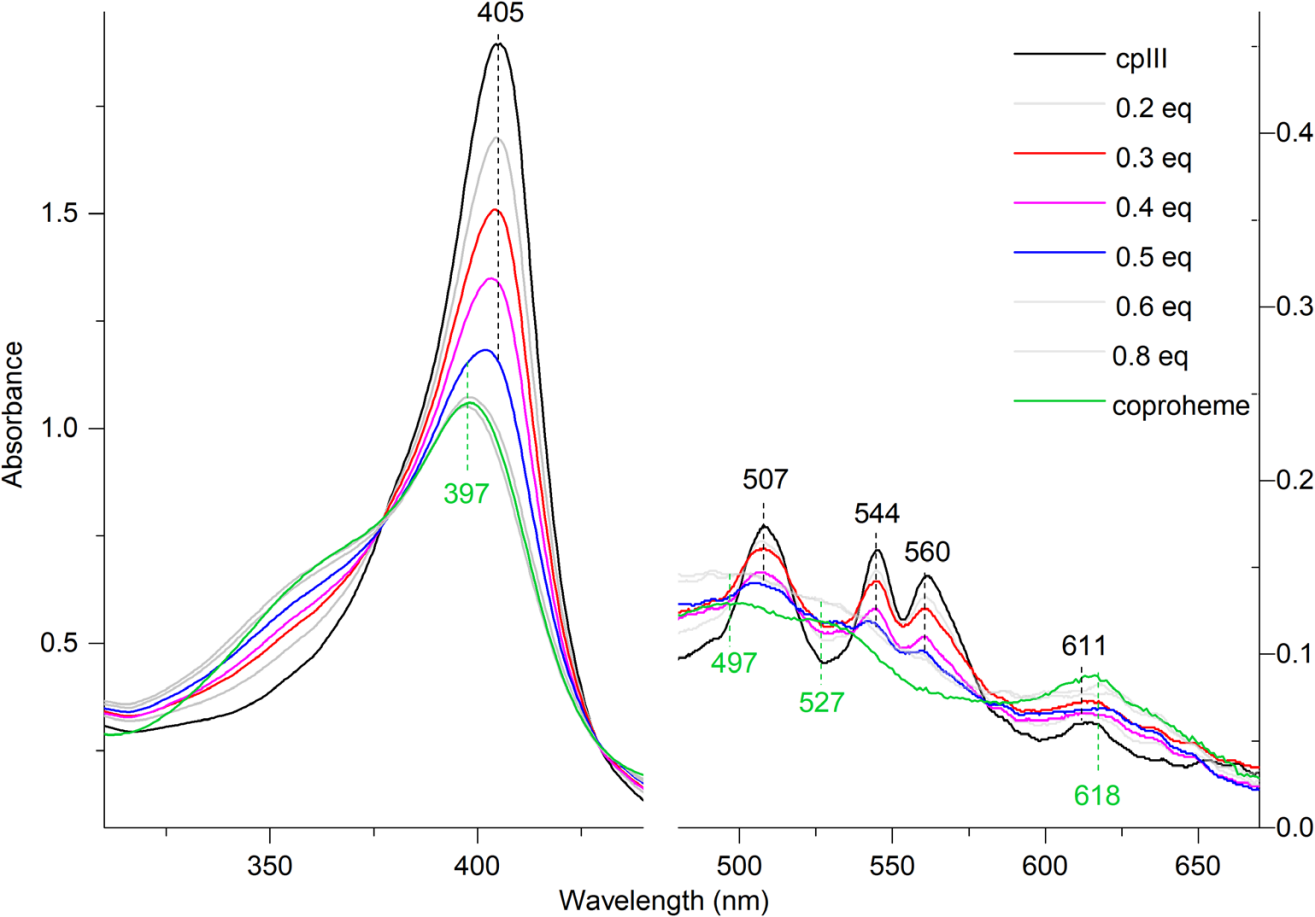
Fe^2+^ in vitro titration under anaerobic conditions of WT *Lm*CpfC‐cpIII solution, followed by UV–vis spectroscopy. The initial spectrum (WT *Lm*CpfC‐cpIII) is reported in black, while the final spectrum (WT *Lm*CpfC‐coproheme) in green, and the titrated solutions with 0.3, 0.4, and 0.5 eq of iron in red, magenta, and blue, respectively. The spectra at other steps of the titration are represented as gray lines. The band wavelengths typical of the WT–cpIII and ‐coproheme complexes are indicated in black and green, respectively.

Based on the overall spectroscopic differences, we were able to follow the ferrous iron titration, under anaerobic conditions, of the WT *Lm*CpfC‐cpIII complex to the formation of the WT *Lm*CpfC‐coproheme complex using both UV–vis absorption and RR spectroscopies. Upon progressive addition of small aliquots (0.1 eq) of Fe^2+^ to the WT‐cpIII complex, the UV‐vis spectra (Figure 3 and Figure S2) change from that typical of WT‐cpIII (bands at 405, 507, 544, 560, and 611 nm) (black spectrum) to the final WT‐coproheme complex spectrum (bands at 397, 497, 527, and 618 nm) (green spectrum) (Dali et al., 2023; Gabler et al., 2022). The result can be interpreted in terms of different equilibrium populations between the WT *Lm*CpfC‐cpIII and WT *Lm*CpfC‐coproheme species during titration. Unexpectedly, unlike in the UV–vis spectra, upon titration, the RR spectra are more difficult to analyze and possibly show the presence of intermediates. In the following, we describe the RR spectroscopy results in the different spectral ranges of interest.

**FIGURE 4:**
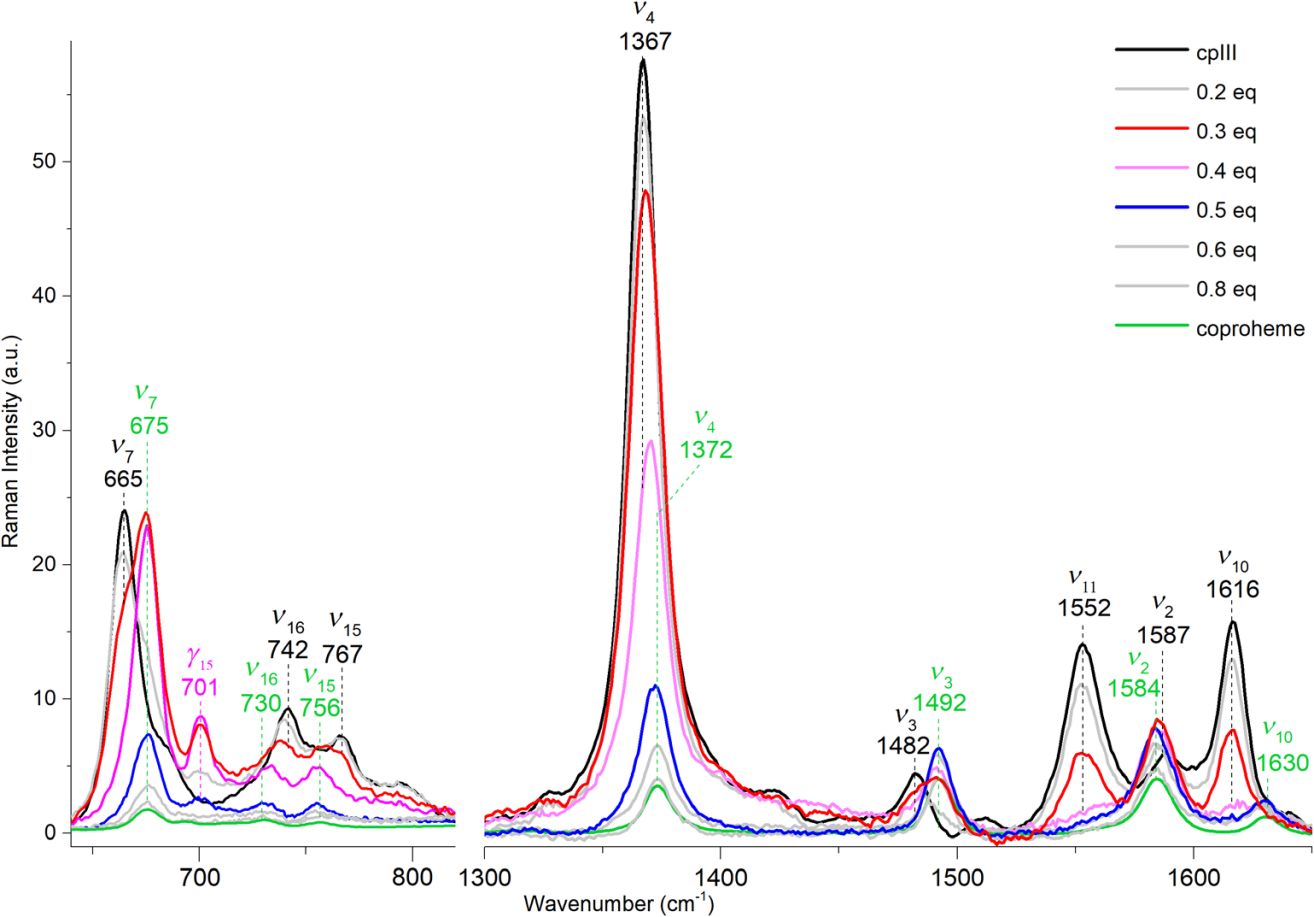
RR spectra in the 640–820 (left) and 1300–1650 cm^−1^ (right) regions of the Fe^2+^ in vitro titration under anaerobic conditions of WT *Lm*CpfC‐cpIII solution. The initial spectrum (WT *Lm*CpfC‐cpIII) is reported in black, the final spectrum (WT *Lm*CpfC‐coproheme) in green, and the titrated solutions with 0.3, 0.4, 0.5 eq of iron in red, magenta, and blue, respectively. The spectra at other steps of the titration are represented as gray lines. The RR out‐of‐plane band wavenumbers are indicated in magenta, and in black and green are indicated the band wavenumbers typical of the WT–cpIII and ‐coproheme complexes, respectively.

*1300–1650 cm* ^*−1*^ *region*: as shown in Figure 4 (right) and Figure S3, the formation of ferric coproheme upon metalation of cpIII, can be easily traced by the change of the core size marker bands from being characteristic of the WT‐cpIII complex to those of the ferric WT‐coproheme complex (e.g., the *ν*_4_ band shifts from 1367 to 1372 cm^−1^). Bound coproheme is clearly revealed on the addition of sub‐stoichiometric amounts of iron to the WT–cpIII complex, by the progressive appearance of the bands at 1492, 1584, and 1630 cm^−1^, assigned to the *ν*_3_, *ν*_2_, and *ν*_10_ modes of the WT–coproheme complex, respectively, with the concomitant decrease of the WT‐cpIII complex bands (at 1482, 1552, 1587, and 1616 cm^−1^, assigned to the *ν*_3_, *ν*_11_, *ν*_2_, and *ν*_10_ modes, respectively). However, the intensity change is not linear with the Fe^2+^ addition. Upon addition of 0.8 eq of Fe^2+^ the spectrum becomes very similar to the WT‐coproheme complex only.

Ferrochelatases catalyze the insertion of ferrous iron into a porphyrin macrocycle. However, adding progressively small aliquots of Fe^2+^ to the WT‐cpIII complex under anaerobic conditions, we are unable to observe the formation of ferrous coproheme. In fact, the ferrous species is characterized by a very different RR spectrum with core size marker bands typical of a mixture of a 5cHS and a 4cIS (Figure S4) (Andersson et al., 1989). We readily observe the core size marker bands typical of 5cHS ferric coproheme, that is, the oxidation occurs in a timescale faster than that of our spectroscopic measurements.

*640–820 cm* ^*−1*^ *region*: similarly to the core size marker band region, bound coproheme is also clearly revealed in this region by the intensity increase of the bands at 675, 730, and 756 cm^−1^ (*ν*_7_, *ν*_16_, and *ν*_15_, modes) at the expense of the corresponding bands of the WT‐cpIII complex (at 665, 742, and 767 cm^−1^). However, in this region (Figure 4, left) it is worth noting the activation of the *γ*_15_ (B_2u_) out‐of‐plane mode at 701 cm^−1^ upon addition of 0.2 eq of iron. Its intensity keeps increasing up to 0.4 eq of Fe^2+^ (magenta line). Concomitantly, an anomalous intensity increase of the *ν*_7_ coproheme mode at 675 cm^−1^ is observed. At 0.5 eq of iron (blue line), the intensity of both the *γ*_15_ and *ν*_7_ bands decreases, and above 0.5 eq of the metal, only the bands typical of the WT–coproheme complex can be observed until the complete formation of the final product.

**FIGURE 5:**
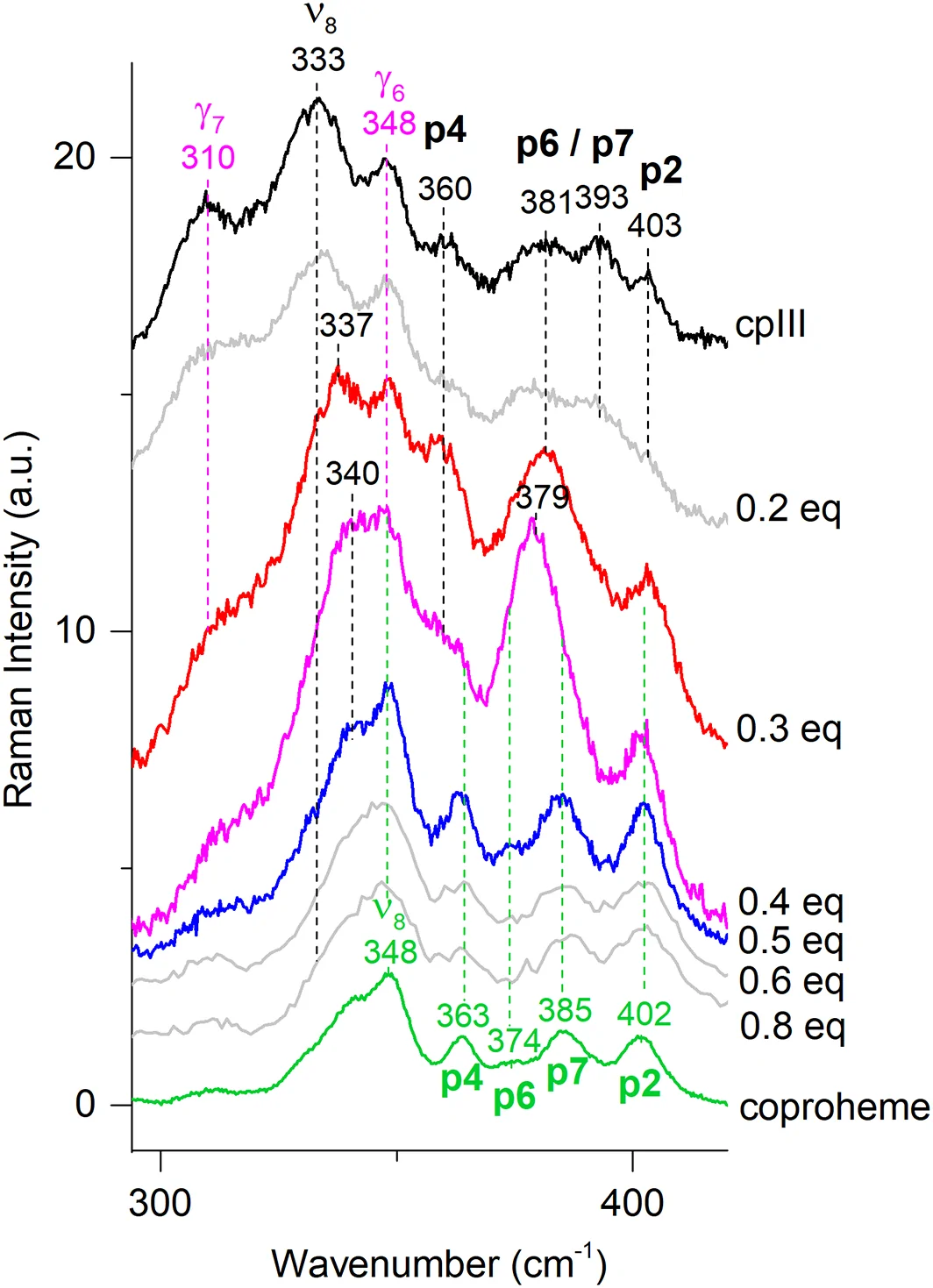
RR spectra in the 290–420 cm^−1^ region of the Fe^2+^ in vitro titration under anaerobic conditions of WT *Lm*CpfC‐cpIII solution. The initial spectrum (WT *Lm*CpfC‐cpIII) is reported in black (top), the final spectrum (WT *Lm*CpfC‐coproheme) in green, and the titrated solutions with 0.3, 0.4, and 0.5 eq of iron in red, magenta, and blue, respectively. The spectra at other steps of the titration are represented as gray lines. The RR out‐of‐plane band wavenumbers are reported in magenta, and in black and green are indicated the band wavenumbers typical of the WT‐cpIII and ‐coproheme complexes, respectively. The spectra have been shifted along the vertical axis for a better visualization.

*290*–*420 cm* ^*−1*^ *region*: titration markedly alters the overall spectra in this region (Figure 5). The *γ*_6_ and *γ*_7_ bands (at 310 and 348 cm^−1^, respectively) characteristic only of the doming‐like distorted WT cpIII‐complex start decreasing in intensity at the very beginning of the Fe^2+^ titration, and at 0.5 eq of metal (blue line), they have almost disappeared. However, the major changes are observed in the wavenumbers and relative intensities of the propionate bending modes δ(C_β_C_c_C_d_), as a consequence of the re‐adjustment of the propionate H‐bonds with the protein, passing from those characteristic of the cpIII complex (360, 381, 393, and 403 cm^−1^) to those of the coproheme complex (363, 374, 385, and 402 cm^−1^) (Figure 5). The band at 403 cm^−1^ assigned to the propionate at position 2 (p2), decreases in intensity at the first step of the titration (0.2 eq). Interestingly, this RR spectrum is very similar to that of the Y124F variant‐cpIII complex, where the hydrogen bond between p2 and the Y124 residue was removed (Dali et al., 2023). The propionate bending mode appears again after the addition of 0.3 eq of Fe^2+^ (red line) giving rise to a band at 402 cm^−1^ whose intensity increases upon titration to the final product. These data suggest that p2 and its H‐bond interactions are a very sensitive probe for metal insertion in the cpIII complex.

Changes of the p4 are difficult to follow due to the weakness of the band at 360 cm^−1^ in the WT‐cpIII complex. However, upon addition of 0.4 eq of ferrous iron (magenta line), a broad band due to the coexistence of cpIII and coproheme can be easily observed, which shifts to 363 cm^−1^ at 0.5 eq of Fe^2+^ (blue line).

Due to a strong coupling between p6 and p7, we were unable to reach a conclusive selective assignment of the bands at 381 and 393 cm^−1^, which were ascribed to their bending modes in the WT–cpIII complex (Dali et al., 2023) The band at 393 cm^−1^ decreases its intensity at the first step of the titration (0.2 eq), and with subsequent additions of the metal it slowly downshifts. At 0.3 (red line) and 0.4 (magenta line) eq of the metal, the p6 and p7 bending modes overlap resulting in the intense band at 379 cm^−1^. Finally, upon addition of 0.5 eq (blue line) of Fe^2+^, all the propionate bands have the characteristic wavenumbers observed in the coproheme complex. Thus, the propionate groups have reached the arrangement observed in the product, even if the coproheme formation is not completed. This is clear from the relative intensity of the *ν*_3_/*ν*_4_ core size marker band in the 1330–1650 cm^−1^ region spectra (Figure 4 right, bottom spectra).

Clearly, in the 290–420 cm^−1^ and 640–820 cm^−1^ wavenumber regions the spectra obtained upon addition of 0.3 (red line) and 0.4 (magenta line) eq of ferrous iron cannot be interpreted as a linear superposition of equilibrium populations of the WT *Lm*CpfC‐cpIII and ‐coproheme. The data strongly suggest the presence of a ferric saddled intermediate species, characterized by the presence of an intense *γ*_15_ out‐of‐plane mode at 701 cm^−1^, whose propionate H‐bonds do not correspond either to the substrate or to the product.

## 3 DISCUSSION

Structural insights into an enzymatic reaction at the molecular level are necessary for understanding the catalytic reaction mechanism. However, due to high reactivity and intermediate species, structural information on enzymes must often be obtained by using model substrates or inhibited/inactivated proteins. In this work, we were able to investigate the insertion of ferrous iron into cpIII mediated by coproporphyrin ferrochelatase from *L. monocytogenes* (*Lm*CpfC) using the physiological substrate, obtaining highly valuable results as neither analogies nor assumptions were necessary. We report three crystal structures showing different iron content during metalation in *Lm*CpfC, identifying the presence and defining the extent of the porphyrin distortions during iron insertion. Moreover, of particular importance, the whole picture of the metalation process in solution has been obtained by measuring the RR spectra during the titration.

One would expect that, during the Fe^2+^ titration in solution, the observed spectra could be simply interpreted in terms of an equilibrium of different populations of the WT‐cpIII and ‐coproheme complexes. However, while the UV–vis spectra and the RR core size marker bands are at least partially consistent with this expectation, upon titration, the RR spectra in the 290–420 and 640–820 cm^−1^ regions are characterized by a marked readjustment of the propionate groups of the porphyrin and by the presence of a new *γ*_15_ out‐of‐plane mode at 701 cm^−1^, not detected either in the WT–cpIII or in the coproheme complexes. This latter band is observed between 0.2 and 0.5 eq of Fe^2+^, reaching its maximum intensity upon addition of 0.4 eq of the metal (Figures 3 and 4). Since its activation is an indication of a saddling (B_2u_) deformation, these data suggest the formation of a stable intermediate species, consisting of a distorted porphyrin macrocycle in equilibrium with the substrate (cpIII) and the product (coproheme). These data agree with the observed structures representing fractions of iron in cpIII of "24% Fe", "34% Fe", and "68% Fe" (Figures 1 and 2), where the structure between 0.3 and 0.4 eq of the metal shows the largest porphyrin distortion. It is noteworthy that most of the distortion originates from the out‐of‐plane characteristics of pyrrole ring A, with the p2 substituent. H‐bonding interactions of p2 with Y124 are unique in CpfCs and were shown to be of major importance for structural integrity and correct cpIII and coproheme binding within the active site (Dali et al., 2023; Gabler et al., 2022).

Distortion of the macrocycle is one of the factors that controls the chemical properties of porphyrins in living organisms (Barkigia et al., 1998; Shelnutt & Medforth, 1998). A saddling deformation represents the appropriate arrangement for metal insertion by exposing both the protons and the lone pairs of the nitrogen atoms of the porphyrin to the incoming ferrous iron, allowing the formation of the first metal–porphyrin bond. The metal insertion leads to the formation of a complex in which the iron atom should be coordinated with two of the nitrogen atoms of coproporphyrin III, while the other two are still protonated. Subsequent deprotonation of the two remaining pyrrole nitrogen atoms ends with the formation of ferric coproheme (Karlberg et al., 2008; Wu et al., 2001).

In solution, the binding of the free‐base coproporphyrin ring into the protein results in a doming distortion as a consequence of the hydrogen bond interactions between the propionates and the polar residues in the active site, while in the crystal phase the free‐base porphyrin ring shows a saddling distortion (Dali et al., 2023). However, during metalation the saddling distortion becomes predominant both in the crystal and in solution. In solution, a change from doming to saddling evidently occurs as a consequence of the propionate hydrogen bond alterations after the iron insertion and oxidation. In both the *Lm*CpfC‐cpIII and ‐coproheme complexes, the porphyrin rings are stabilized by the formation of H‐bonds of different strength between polar amino acids of the protein pocket and the propionate groups of the porphyrin. While all the four propionate side‐chain interactions are an essential requirement for the correct orientation and stabilization of the cpIII within the active site (Dali et al., 2023), for the *Lm*CpfC‐coproheme complex the interactions with p2 and p4 were found to be dominant (Gabler et al., 2022). At the early stages of the metal titration of the *Lm*CpfC‐cpIII complex, the RR spectra highlight the relevant changes in the band associated with the propionate p2 conformation, which corresponds to that characteristic of coproheme in the final orientation. However, both in solution and in the crystal, the hydrogen bond interactions involving p6 and p7 are the most affected by the iron insertion. Indeed, in solution the bending modes assigned to p6 and p7 show significant changes in their relative intensities and wavenumbers during metalation, which, to a certain extent, are supported by the x‐ray structures. In the crystal the R45 H‐bonding distance to p6 changes during titration, due to the different conformation adopted by this residue upon iron insertion (Figure 2a). Moreover, the R29–p7 interaction also undergoes major changes during titration, as a consequence of variations in the R29 orientation. The residue points toward p7 in the “24% Fe”, “34% Fe”, and in the coproheme‐complex structures, but points away (5.4 Å) in the “68% Fe” structure (Figure 2b).

Once the propionates have established the interactions typical of the coproheme complex the distortion slowly decreases, to reach the final product, which is almost planar, as indicated by the absence of any out‐of‐plane modes in its RR spectrum. In agreement with the x‐ray crystal structures of human and yeast ferrochelatases, also in a bacterial ferrochelatase the catalysis depends on the iron binding to a saddle‐distorted porphyrin substrate and the release of a flat, metalated product. The present experimental data are consistent with the metalation mechanism suggested by Al‐Karadaghi and coworkers (Al‐Karadaghi et al., 2006). These authors proposed that first a saddled distortion of the tetrapyrrole porphyrin upon binding to the enzyme occurs, followed by the metal‐porphyrin bonding and leading to the formation of an iron saddled complex in which two pyrrole nitrogen atoms coordinate to the metal ion and two protons remain on the pyrrole nitrogen atoms. The sequential deprotonation of the two pyrrole nitrogen atoms leads to the final flat coproheme product. Our results clearly show the existence of saddled, stable, intermediate species containing a ferric ion atom, separated by an energy barrier from the subsequent deprotonated species.

One major open question concerning the reaction mechanism of ferrochelatases (both PpfCs and CpfCs) is the site of the entry channel of the ferrous iron. In other words, once the porphyrin substrate is bound, does the ferrous iron approach from the proximal or the distal side? This issue has been debated for a long time and the consensus became that the metal is inserted from the distal porphyrin side, as there is structural evidence showing a ferrous atom bound by the distal histidine and glutamate residues in CpfC from *Bacillus subtilis (*Hansson et al., 2007 *)*. This structure was obtained by soaking apo‐*Bs*CpfC with Fe^2+^, but no porphyrin was present in that experiment. Also, other divalent cations (e.g., Zn^2+^, Cd^2+^) were proposed to bind at this site (Lecerof et al., 2003). These results were also supported by mutational and mechanistic data on yeast, murine and human PpfCs (Ferreira et al., 2002; Karlberg et al., 2002; Sellers et al., 2001). However, more recent experiments on human ferrochelatase indicate that Fe^2+^ approaches from the proximal side, in agreement with computational studies (Medlock et al., 2021; Wang & Shen, 2013; Wu et al., 2016). Whether this conclusion also holds true for bacterial ferrochelatases, containing a proximal tyrosine ligand and complexed with their physiological substrate, is not clear yet. In this regard, of particular interest is the report that iron‐mediated substrate inhibition could be overcome by manipulating a glutamate residue on the distal side of the heme in CpfC from *Staphylococcus aureus* (Hobbs et al., 2017).

By examining the crystal structures in the present work, at first glance it appears that the ferrous iron approaches from the side of the proximal tyrosine (Y12). Nevertheless, a definitive conclusion cannot be drawn as the ionic radius of the ferrous iron in the “24% Fe”, “34% Fe”, and “68% Fe” structures is larger than the ionic radius of the ferric ion in the coproheme product and would result in an out‐of‐plane position regardless of the side from which it is approaching. Interestingly, in the “68% Fe” structure, we observe a continuous electron density from the distal histidine (H182) to the proximal tyrosine (Y12) (Figure 2a). Computational and experimental studies are underway in order to answer this important mechanistic riddle. Possibly, phylogenetically distinct ferrochelatases may exhibit different behavior in this regard with the specific active site architecture being the factor that determines from which side of the heme the ferrous iron approaches.

## 4 CONCLUSION

Porphyrin distortion has long been discussed to play an essential role in iron insertion of ferrochelatase enzymes. In this study we were able to follow the metalation process using the physiological substrates (coproporphyrin III and ferrous iron) of the coproporphyrin ferrochelatase from *L. monocytogenes* bacterium. The metalation mechanism includes a porphyrin saddling distortion, which becomes predominant both in the crystal and in solution at iron to cpIII ratios between 0.3 and 0.4. This stable and distorted intermediate species results from the readjustment of hydrogen bond interactions of the coproporphyrin III propionates with the protein during the enzymatic catalysis. The distortion slowly decreases upon further addition of iron, as the propionates establish the interactions typical of the almost planar coproheme complex product.

## 5 MATERIALS AND METHODS

### 5.1 X‐ray diffraction

#### 5.1.1 Crystallization and soaking

Crystals of WT *Lm*CpfC in complex with cpIII were obtained in 17.455% (w/v) PEG MME 2 K, 0.1 M BIS‐TRIS pH 6.3 and 0.2 M calcium acetate, as previously described (Dali et al., 2023). To soak the crystals with Fe^2+^, a solution was prepared to match the crystallization conditions, containing 1 mM Fe^2+^ and 20% glycerol as a cryo‐protectant. To prevent unwanted iron oxidation prior to soaking, Fe^2+^ was added to the soaking solution shortly before the soaking procedure. The 25 mM Fe^2+^ stock solution was freshly prepared in a Whitley DG250 Anaerobic Workstation (Don Whitley Scientific, Bingley, UK) under anaerobic conditions. Crystals were soaked under the microscope (SteREO Discovery.V12, Zeiss, Germany) by adding 2 μL of soaking solution to the 300 nL drop containing the crystals and mixing very gently with the pipette. After mixing, the crystals were removed from the soaking solution after 2, 3, and 4 min, and immediately flash‐vitrified in liquid nitrogen.

#### 5.1.2 Data collection and refinement

Data sets were collected at the European Synchrotron Radiation Facility (Grenoble, France) at the beamline ID‐30B at 100 K using a DECTRIS PILATUS 6M detector (doi: 10.15151/ESRF‐ES‐771371625 and 10.15151/ESRF‐ES‐1022127512). Both data sets of crystals soaked for 2 and 4 min were collected using a beam wavelength of 0.97 Å and processed using the EDNA_proc pipeline. The data set for the crystal soaked for 3 min was collected at 1.7 Å beam wavelength. Xtriage was used to evaluate data quality and the pdb structure of *Lm*CpfC wild‐type in complex with coproporphyrin III (PDB ID: 8AT8) to solve the phase problem by molecular replacement with Phaser‐MR (McCoy et al., 2007). With AUTOBUILD (Terwilliger et al., 2008) the initial models were generated and further improved by iterative cycles of manual model generation with COOT (Emsley et al., 2010), as well as maximum likelihood refinement with PHENIX‐refine (Adams et al., 2010). The French and Wilson algorithm (French & Wilson, 1978) was applied by PHENIX‐refine to transform intensities to amplitudes. The refinements were completed applying automatic addition of hydrogen and water molecules, translation liberation screw (TLS), optimization of x‐ray/ADP weight, and optimization of isotropic B‐factor model for x‐ray/stereochemical weight. The models were validated using MolProbity (Chen et al., 2010) and images were created using PyMOL (http://www.pymol.org). The chemical structure file (MOL file) of coproporphyrin III (ChEBI ID: 27609) from the Chemical Entities of Biological Interest (ChEBI) was used as a template in CCP4 (within the Make Ligand—AceDRG application (Agirre et al., 2023; Debreczeni & Emsley, 2012; Long et al., 2017)) to generate the crystallographic information file (.cif) for coproporphyrin III needed in the structural refinements process done by the PHENIX‐refine program.

### 5.2 Distortion degree analysis in PyMOL

Analysis of distortion was done by PyMOL (by Schrödinger, www.pymol.org). The *Lm*CpfC‐coproheme structure (PDB‐ID: 6SV3) was superimposed to the *Lm*CpfC‐cpIII (PDB‐ID: 8AT8) and each Fe‐soaked structures (PDB‐IDs: 8BBV; 8OMM; and 8OFL). First, pyrrole rings A and B of the corresponding structures were pair‐fitted using the corresponding pyrrole nitrogens and the angles with respect to the coproheme pyrrole rings were measured. The process was repeated analogously for pyrrole rings C and D.

### 5.3 UV–vis and RR spectroscopy

#### 5.3.1 Sample preparation

CpIII was purchased from Frontiers Scientific (product number: C654‐3) as lyophilized powder and dissolved in 0.5 M NaOH. The WT *Lm*CpfC‐cpIII complex was prepared by adding the cpIII solution to WT *Lm*CpfC apoprotein dissolved in 50 mM HEPES buffer, pH 7.4. We used a cpIII: apoprotein ratio of 1:1.3 to ensure the complete binding of the substrate to the protein (Dali et al., 2023).

The iron titration in vitro was carried out inside a glovebox workstation (LABstar, MBRAUN, Germany), with an oxygen concentration below 0.5 ppm, using an iron (II) sulfate heptahydrate (Sigma Aldrich, St. Louis, MO) solution in 50 mM HEPES buffer, pH 7.4, previously degassed by flushing with nitrogen gas. We progressively added the Fe^2+^ solution in small steps (0.1 eq) to a previously degassed WT *Lm*CpfC‐cpIII complex solution under continuous agitation (with a magnetic stirrer). To reach equilibrium at each stage of the iron titration, each sample was anaerobically transferred and sealed, inside the glovebox, into a 5 mm NMR tube after 3 min from preparation. UV–Vis and RR spectra have been obtained on these samples and no spectral changes have been observed within an hour from preparation. Ferric coproheme was purchased from Frontier Scientific, Inc. (Logan, UT) as lyophilized powder. The ferric WT *Lm*CpfC‐coproheme complex was prepared by adding the coproheme alkaline solution (i.e., dissolved in 0.5 M NaOH) to a solution of the WT *Lm*CpfC apoprotein dissolved in 50 mM HEPES buffer, pH 7.4. We used a coproheme: apoprotein ratio of 1:1.2 to ensure the complete binding of the product to the protein (Gabler et al., 2022). The ferrous WT *Lm*CpfC‐coproheme complex was prepared by flushing the ferric WT *Lm*CpfC‐coproheme complex solution with nitrogen gas and, then, by reducing it with a freshly prepared sodium dithionite solution (20 mg mL^−1^).

Sample concentration, in the range 35–60 μM for both the UV–vis and RR experiments, was determined using an extinction coefficient of 101,006 M^−1^ cm^−1^ (coproheme) and 93,594 M^−1^ cm^−1^ (cpIII) at 393 nm (Dali et al., 2023). No changes in the RR spectra have been observed between pH 6.3 and 7.4.

#### 5.3.2 UV–vis electronic absorption spectroscopy

UV–vis electronic absorption spectra were obtained at room temperature using a 1 cm cuvette or a 5 mm NMR tube by means of a Cary 60 spectrophotometer (Agilent Technologies, Santa Clara, CA) with a resolution of 1.5 nm and a scan rate of 300 nm min^−1^. For the calculation of the second derivative, the Savitzky–Golay method was applied to the spectra using 15 data points and a third order degree polynomial function (LabCalc, Galactic Industries, Salem, NH). No significative differences in the wavelength or in the bandwidth were observed, when the number of points was changed. To ensure that no degradation of the sample occurred under the experimental conditions used, the UV–vis electronic absorption spectra were recorded both before and after RR measurements. All the spectra were normalized to the concentration of the sample.

#### 5.3.3 RR spectroscopy

The RR spectra were obtained at room temperature by placing the samples in a slowly rotating 5 mm NMR tube and using the 413.1 nm laser line of an Innova300C Kr^+^ laser (Coherent, Santa Clara, CA). Back‐scattered light was collected and focused into a triple spectrometer (Acton Research, Acton, MA), consisting of two SpectraPro 2300i instruments working in the subtractive mode and, in the final stage, a SpectraPro 2500i instrument equipped with a grating of 3600 or 1800 grooves mm^−1^ and a liquid nitrogen‐cooled charge‐coupled device (CCD) detector (Roper Scientific Princeton Instruments). The laser power at the sample was 1 mW for all the samples.

Spectral resolutions of 1.2 cm^−1^ for the grating of 3600 grooves mm^−1^, and 4 cm^−1^ for the grating of 1800 grooves mm^−1^ were calculated based on the optical properties of the spectrometer. The RR spectra were calibrated using indene and carbon tetrachloride as standards to an accuracy of 1 cm^−1^ for intense isolated bands. All the RR measurements were repeated several times and summed, if no spectral differences were noted, to ensure reproducibility and to improve the signal‐to‐noise ratio. Table S1 summarizes the integration time and the number of averaged spectra reported in the figures. No evidence was found for a time evolution of the RR spectra over the experimental time windows (minutes to hours). Therefore, the RR data are taken as measurements of the properties of a system under thermodynamic equilibrium. All the spectra reported were baseline‐corrected, normalized to the concentration of the sample and the integration time.

## Supporting information


**Data S1:** Supporting Information
