# Supplementary material for: Iron insertion into coproporphyrin III‐ferrochelatase complex: Evidence for an intermediate distorted catalytic species
Source: Protein Sci. 2023 Nov 1;32(11):e4788. doi: 10.1002/pro.4788 (PMC10578119; doi:10.1002/pro.4788)
Supplement: Supplementary file 1 — Data S1: Supporting Information [file PRO-32-e4788-s001.docx]

Supporting Information

**Iron insertion into the coproporphyrin III-ferrochelatase complex: evidence for an intermediate distorted catalytic species**

Thomas Gabler^$1^, Andrea Dali^$2^, Federico Sebastiani^$2^, Paul Georg Furtmüller^1^, Maurizio Becucci^2*,^ Stefan Hofbauer^1^* and Giulietta Smulevich^2,3^*

^1^University of Natural Resources and Life Sciences, Vienna, Department of Chemistry, Institute of Biochemistry, Muthgasse 18, A-1190 Vienna, Austria

^2^Dipartimento di Chimica “Ugo Schiff” (DICUS), Università di Firenze, Via della Lastruccia 3-13, I-50019 Sesto Fiorentino (FI), Italy

^3^INSTM Research Unit of Firenze, Sesto Fiorentino (Fi), Italy

^$^ These authors contributed equally

Corresponding Authors

*e-mail: Maurizio.becucci@unifi.it, phone: +39-055-4573089

*e-mail: stefan.hofbauer@boku.ac.at, phone: +43-1-47654-77258, fax: +43-1-47654-77059

*e-mail: giulietta.smulevich@unifi.it, phone: +39-055-4573083

**Running title**: Structural studies of iron insertion by CpfC

**Table S1**. Integration time and number of averaged RR spectra of WT *Lm*CpfC-cpIII, -coproheme ferric and ferrous complex and upon Fe^2+^ titration, obtained at low (grating: 1800 grooves/mm) and high resolution (grating: 3600 grooves/mm).

| Sample | Low resolution  (**Figures 4**, **S2** and **S3**) | High resolution  (**Figure 5**) |
| --- | --- | --- |
| WT - cpIII | 110 spectra / 110 min | 50 spectra / 250 min |
| WT- coproheme Fe(III) | 10 spectra / 25 min | 8 spectra / 60 min |
| WT - coproheme Fe(II) | 8 spectra / 40 min | - |
| WT - cpIII + 0.2 eq of iron | 20 spectra / 20 min | 15 spectra / 60 min |
| WT - cpIII + 0.3 eq of iron | 30 spectra / 60 min | 35 spectra / 70 min |
| WT - cpIII + 0.4 eq of iron | 10 spectra / 10 min | 35 spectra / 70 min |
| WT - cpIII + 0.5 eq of iron | 20 spectra / 20 min | 45 spectra / 90 min |
| WT - cpIII + 0.6 eq of iron | 20 spectra / 20 min | *see Low Resolution* |
| WT - cpIII + 0.8 eq of iron | 30 spectra / 30 min | *see Low Resolution* |

**Figure S1.** Pair-fitting analysis of porphyrin distortions (in degrees) of coproporphyrin III *versus* coproheme. The *Lm*CpfC – coproheme structure (PDB-ID: 6SV3) was superimposed on that of *Lm*CpfC – cpIII (PDB-ID: 8AT8) and each Fe – soaked structure (PDB-IDs: 8BBV, 2min; 8OMM, 3 min, and 8OFL, 4 min). First, pyrrole rings A and B of the corresponding structures were pair-fitted using the corresponding pyrrole nitrogens then the angles with respect to the coproheme pyrrole rings were measured. The process was repeated for pyrrole rings C and D. After alignment, the angle difference of each pyrrole ring (black - A, red - B, green - C, blue – D) was measured and plotted against the percent of iron insertion, determined in the various structures labeled according to their pdb code. The pink dots are the sum of the absolute values of each angle for the four pyrrole rings. The analysis shows how the angles of all four pyrrole rings change starting from the *Lm*CpfC structure (PDB-ID: (AT8) (substrate), upon insertion of iron in the three newly solved structures (PDB-ID: 8BBV, 24% Fe, 8OMM 34% Fe, 8OFL 67% Fe), as compared to the -*Lm*CpfC- coproheme structure (PDB-ID: 6SV3) (product).

**

**

**Figure S2**. Fe^2+^ in vitro titration under anaerobic conditions of WT *Lm*CpfC – cpIII solution, followed by UV–vis spectroscopy. The initial spectrum (WT *Lm*CpfC – cpIII) is reported in black (top), the final spectrum (WT *Lm*CpfC – coproheme) in green (bottom), and the titrated solutions with 0.3, 0.4, 0.5 eq of iron in red, magenta and blue, respectively. The spectra at other steps of the titration are represented as grey lines. The band wavelengths typical of the WT – cpIII and – coproheme complexes are indicated in black and green, respectively. The spectra have been shifted along the vertical axis for a better visualization.





**Figure S3.** RR spectra in the 640-820 (left) and 1300-1650 cm^-1^ (right) regions of the Fe^2+^ in vitro titration under anaerobic conditions of WT *Lm*CpfC-cpIII solution. The initial spectrum (WT *Lm*CpfC–cpIII) is reported in black (top), the final spectrum (WT *Lm*CpfC–coproheme) in green (bottom), and the titrated solutions with 0.3, 0.4, 0.5 eq of iron in red, magenta, and blue, respectively. The spectra at other steps of the titration are represented as grey lines. The RR out-of-plane band wavenumbers are indicated in magenta, while in black and green are indicated the band wavenumbers typical of the WT–cpIII and –coproheme complexes, respectively. The spectra have been shifted along the vertical axis for a better visualization.





**Figure S4**. Comparison of the UV-Vis electronic absorption (left) and RR spectra in the 1300-1650 cm^-1^ region (right) of ferric and ferrous WT *Lm*CpfC-coproheme complexes. The wavelengths and the RR band wavenumbers are reported in green and orange for the ferric and ferrous complexes, respectively. The ferrous form is a mixture of a 5cHS and a 4cIS species (underlined bands at 1594 cm^-1^, ν_2_, and 1641 cm^-1^, ν_10_) (Andersson, L. A.; Mylrajan, M.; Sullivan, E. P., Jr.; Strauss, S. H., Modeling low-pH hemoproteins. *J Biol Chem* 1989, *264* (32), 19099-102).
